# Assessment of Antibiotic Prescriptions for Lyme Disease After Modification of Reporting Language for Positive Screening Test Results

**DOI:** 10.1001/jamanetworkopen.2021.44928

**Published:** 2022-01-25

**Authors:** Sarah J. Willis, Noelle M. Cocoros, Myfanwy Callahan, Brian Herrick, Catherine M. Brown, Benjamin A. Kruskal, Michael Klompas

**Affiliations:** 1 Department of Population Medicine, Harvard Medical School and Harvard Pilgrim Health Care Institute, Boston, Massachusetts; 2 Atrius Health, Boston, Massachusetts; 3 Cambridge Health Alliance, Cambridge, Massachusetts; 4 Bureau of Infectious Disease and Laboratory Sciences, Massachusetts Department of Public Health, Boston, Massachusetts; 5 New England Quality Care Alliance, Braintree, Massachusetts; 6 Department of Medicine, Brigham and Women’s Hospital, Boston, Massachusetts

## Abstract

This quality improvement study assesses whether revision of positive Lyme disease test result text was associated with decreases in the frequency of antibiotic prescriptions for patients without confirmatory results.

## Introduction

Approximately 476 000 individuals are treated for Lyme disease in the US annually.^1^ However, up to 50% of antibiotics prescribed for Lyme disease may be unnecessary.^2^ Clinicians using the standard 2-tiered testing algorithm may overprescribe because they interpret positive enzyme-linked immunosorbent assay (ELISA) results as evidence of Lyme disease instead of waiting for the more specific Western immunoblot (WB) test to confirm the diagnosis.^3^

To encourage clinicians to wait for confirmatory WB before prescribing antibiotics, a large health care system in Massachusetts revised the reporting of positive Lyme disease ELISA test results in their electronic health record from *positive* to *WesternToFollow* in August 2015, reasoning that language indicating that testing was still in progress might decrease reflexive prescribing behavior. Our objective was to determine whether the revision of positive Lyme disease ELISA result text was associated with decreased frequency of antibiotic prescriptions for patients without confirmatory results.

## Methods

In this quality improvement study, we compared antibiotic prescribing behavior between 2 large practice groups in eastern Massachusetts: Atrius Health, which revised reporting of Lyme disease ELISA test results, and Cambridge Health Alliance (CHA), which did not. Neither practice changed treatment protocols during the study period. Data were accessed via the Electronic Medical Record Support for Public Health surveillance platform.^4^ Data on race and ethnicity were collected during routine clinical care in the electronic medical records (likely self-reported). All patients who reported Hispanic were classified as Hispanic. Patients who did not report Hispanic ethnicity were categorized as non-Hispanic plus reported race. The Harvard Pilgrim Health Care Institute institutional review board determined that this study was non–human participant research.

We selected patients with at least 1 positive Lyme disease ELISA test result between January 1, 2010, and December 31, 2019, and assessed for a positive Lyme disease IgG or Lyme disease IgM WB test result within 30 days of the ELISA collection date. Patients with orders for antibiotics potentially used to treat Lyme disease within 30 days of their positive ELISA test result were considered treated (eMethods in the Supplement).

We performed interrupted time series analyses to compare the percentage of positive Lyme disease ELISA test results treated without a positive confirmatory test before and after Atrius revised the result text. We also estimated how the change at Atrius differed from the change at CHA using a difference-in-difference model. The analysis was conducted at the test level not patient level (some patients have more than one positive test result over time). Additional details on the methods are available in eMethods in the Supplement. A 2-sided *P* < .05 was considered statistically significant. All data analyses were conducted using SAS software, version 9.4 (SAS Institute Inc). This study followed the Standards for Quality Improvement Reporting Excellence (SQUIRE) reporting guideline for quality improvement studies.

## Results

There were 16 015 positive Lyme disease ELISA test results among 13 048 unique patients: 14 986 tests (94%) at Atrius and 1029 tests (6%) at CHA. Slightly more than half of positive ELISA test results (53%) were among female patients. The median age of patients with positive ELISA test results was 43 years (IQR, 20-59 years) at Atrius and 35 years (IQR, 26-47 years) at CHA. Most positive ELISA test results were among non-Hispanic White patients at Atrius (84%) and CHA (52%).

Before the revision of ELISA test result text, 27% of positive ELISA test results at Atrius were treated for Lyme disease without a positive WB result (β = –0.0007; *P* = .63) (Figure). There was an immediate nonsignificant decrease in the percentage of positive ELISA test results treated without a positive WB result after the result text was changed in quarter 3 of 2015 (25% to 19%; β = –0.06; *P* = .05). The percentage of positive ELISA tests treated without a positive confirmatory test result decreased to 8% in quarter 4 of 2019 (β = –0.007; *P* = .01). At CHA, the percentage of positive ELISA test results that were treated without a positive WB result was 13% initially and remained stable. A difference-in-differences model confirmed a significant decrease in positive Lyme disease ELISA test results that were treated without positive WB result at Atrius vs CHA (β = –0.01; *P* = .02).

**Figure.**
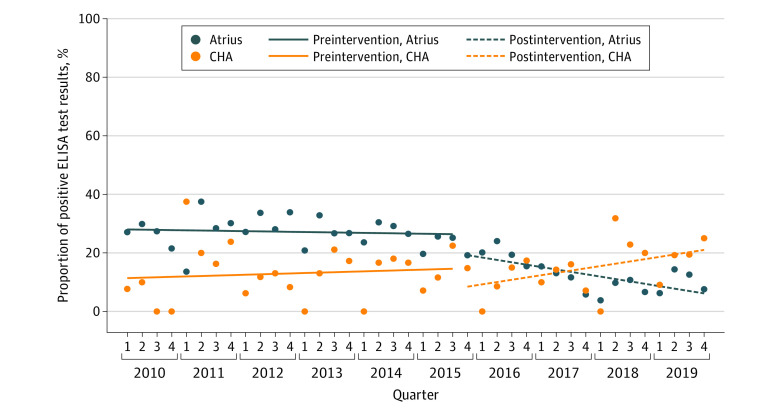
Quarterly Percentages of Positive Lyme ELISA Test Results That Were Treated Without a Positive Confirmatory Western Immunoblot Test, Atrius and CHA, January 2010 through December 2019 Atrius indicates Atrius Health practice group; CHA, Cambridge Health Alliance; ELISA, enzyme-linked immunosorbent assay.

## Discussion

The revised Lyme disease ELISA test result text resulted in a 70% decrease in tests in which the patient was treated without confirmed Lyme disease at a practice using the standard 2-tiered testing algorithm. This simple low-cost intervention may be associated with a substantial reduction in unnecessary antibiotic use for Lyme disease. Overdiagnosis and overtreatment of Lyme disease have been associated with antibiotic adverse effects, expenses, and delays in appropriate diagnosis.^5^ At the population level, antibiotic overuse may accelerate antibiotic resistance.^6^

This study has limitations. First, we did not have clinical information about patients with positive ELISA test results and may have misclassified some appropriate treatments (eg, patients with erythema migrans and a positive ELISA result but negative WB result). Some antibiotics ordered may not have been prescribed for Lyme disease. However, we do not expect the frequency of misclassification to have changed before vs after the intervention. Second, the demographic characteristics and treatment frequency for patients at CHA differed from patients at Atrius. While confounding may explain baseline differences in antibiotic prescribing between the sites, it would not explain the change found at Atrius after their modifications to the reporting language.

The findings of this quality improvement study suggest the potential utility of shifting the way in which test results are reported for changing clinician prescribing behavior.

## Notes

Supplement. **eMethods.** Antibiotics Potentially Used to Treat Lyme Disease
