# Supplementary material for: Assessment of Antibiotic Prescriptions for Lyme Disease After Modification of Reporting Language for Positive Screening Test Results
Source: JAMA Netw Open. 2022 Jan 25;5(1):e2144928. doi: 10.1001/jamanetworkopen.2021.44928 (PMC8790673; doi:10.1001/jamanetworkopen.2021.44928)
Supplement: Supplement. — eMethods. Antibiotics Potentially Used to Treat Lyme Disease [file jamanetwopen-e2144928-s001.pdf]

Supplemental Online Content

Willis SJ, Cocoros NM, Callahan M, et al. Assessment of antibiotic prescriptions for Lyme disease after modification of reporting language for positive screening test results. *JAMA Netw Open*. 2022;5(1):e2144928. doi:10.1001/jamanetworkopen.2021.44928

eMethods. Antibiotics Potentially Used to Treat Lyme Disease

This supplemental material has been provided by the authors to give readers additional information about their work.

© 2022 Willis SJ et al. *JAMA Network Open*.

eMethods. ANTIBIOTICS POTENTIALLY USED TO TREAT LYME DISEASE

Doxycycline, Amoxicillin, Ceftriaxone, Cefuroxime axetil, Cefotaxime, Azithromycin, and Tetracycline

eMETHODS

We assessed the impact of the revised ELISA result text on the percentage of positive Lyme ELISA tests that were treated without a confirmatory test or with a negative confirmatory test. The outcome was measured quarterly from January 1, 2010 through December 31, 2019.

We performed interrupted time series analyses to estimate the effect of the revised result text. Segmented linear regression models were used to compare the percentage of positive ELISAs that were treated without a positive confirmatory test before and after the change in ELISA result text. The baseline period included the first quarter of 2010 through the third quarter of 2015 (time period before and when the ELISA result text was changed) and the intervention period included the fourth quarter of 2015 through the fourth quarter of 2019 (time period after the ELISA result text was changed). We used an autoregressive form of segmented linear regression and tested for the presence of autocorrelation between quarters using the Durbin-Watson test.

The regression model estimated three coefficients: the slope, or average quarterly change in the outcome before the result text was changed; the slope, or average quarterly change in the outcome after the result text was changed; and the change in slope immediately after the result text was changed in quarter 3, 2015.

Lastly, we ran a regression model in which the outcome was the difference in the percentage of positive ELISA tests treated in the absence of a positive confirmatory test between Atrius and CHA every quarter. Taking the difference in percentages allowed us to collapse the two time-series into one and estimate how the change at Atrius differed from the change at CHA, i.e. difference-in-differences.

All data analyses were conducted with SAS version 9.4 (SAS Institute Inc., Cary, North Carolina, US).

© 2022 Willis SJ et al. *JAMA Network Open*.
